# Designing, implementing and testing an intervention of affective intelligent agents in nursing virtual reality teaching simulations—a qualitative study

**DOI:** 10.3389/fdgth.2024.1307817

**Published:** 2024-04-18

**Authors:** Michael Loizou, Sylvester Arnab, Petros Lameras, Thomas Hartley, Fernando Loizides, Praveen Kumar, Dana Sumilo

**Affiliations:** ^1^ Centre for Health Technology, University of Plymouth, Plymouth, United Kingdom; ^2^ Centre for Postdigital Cultures, Coventry University, Coventry, United Kingdom; ^3^ Department of Computer Science, University of Wolverhampton, Wolverhampton, United Kingdom; ^4^ School of Computer Science and Informatics, Cardiff University, Cardiff, United Kingdom; ^5^ School of Health and Social Wellbeing, University of the West of England, Bristol, United Kingdom; ^6^ Warwick Medical School, University of Warwick, Warwick, United Kingdom

**Keywords:** affective, virtual, reality, VR, nursing, intelligent, teaching, simulation

## Abstract

Emotions play an important role in human-computer interaction, but there is limited research on affective and emotional virtual agent design in the area of teaching simulations for healthcare provision. The purpose of this work is twofold: firstly, to describe the process for designing affective intelligent agents that are engaged in automated communications such as person to computer conversations, and secondly to test a bespoke prototype digital intervention which implements such agents. The presented study tests two distinct virtual learning environments, one of which was enhanced with affective virtual patients, with nine 3rd year nursing students specialising in mental health, during their professional practice stage. All (100%) of the participants reported that, when using the enhanced scenario, they experienced a more realistic representation of carer/patient interaction; better recognition of the patients' feelings; recognition and assessment of emotions; a better realisation of how feelings can affect patients' emotional state and how they could better empathise with the patients.

## 1 Introduction

Simulation is an educational tool that is becoming increasingly prevalent in nursing education (1) provides students with realistic clinical situations allowing them to practice and learn in a safe environment (2). Human patient simulators provide experiences that are realistic and offer students an opportunity to assess, intervene, and evaluate patient outcomes (3). Findings from a meta-analysis suggests that simulation education improved nursing students’ performance in clinical reasoning skills (4).

A considerable amount of literature focuses on the use of digitally-mediated tools in healthcare. For example, a systematic review of 12 studies investigated the effectiveness of dementia carer-oriented interventions delivered through the internet and showed improvements in carer wellbeing (5). The review highlighted that, multicomponent individually tailored programmes that combine information, caregiving strategies, and contact with other carers can increase their confidence and self-efficacy, reducing stress and depression. The evolution of nursing education teaching technologies has witnessed a shift towards interactive and immersive learning experiences. Building upon the observation by Baysan et al. (6) regarding the prevalent use of videos, recent studies have explored the integration of virtual reality (VR) and augmented reality (AR) simulations in nursing education. For instance, a study by Nakazawa et al. (7) shows that training with AR is effective in enhancing caregivers’ physical skills and fostering greater empathy towards their patients. This approach not only enhances learner engagement but also facilitates active learning and skill retention.

However, despite the potential benefits of digital health interventions, addressing the emotional challenges and stressors faced by participants remains a critical concern. Research has highlighted the impact of environmental factors and basic emotions on user experience and outcomes in health teaching simulations (8, 9). Another study found undergraduate students felt unready, were anxious about having their mistakes exposed, worried about damaging teamwork and were afraid of evaluation (10).

According to Rippon (9), anger in healthcare environments is a very common emotion, leading to aggression and violence, and healthcare professionals are exposed to it daily with an increasing number of them suffering from signs of post-traumatic stress disorder. Although these problems have been identified and the potential of technologies has been widely recognised, most Human-Computer Interaction applications tend to overlook these emotions when responding to user input (11).

Thus, there is an increasing need for computer systems to endow affective intelligent agents with emotional capabilities and socially intelligent behaviour, and the aim of having an affective interaction between virtual and human users and an effect on the affective state of the user (12–14). This article proposes the design, implementation, and evaluation of a model for incorporating emotional enhancements (concentrating on negative emotions such as stress, fear, and anxiety) into virtual agents applied to virtual teaching applications for healthcare provision. For this purpose, we have created the following research objectives:

• RO1—To what extent does a virtual learning scenario incorporating emotional virtual agents provide a realistic experience.
• RO2—To what extent does the incorporation of emotions into virtual agents stimulate a better set of responses from the human user.
• RO3—To what extent do emotional virtual agents improve the learning experience.
• RO4—To what extent do emotional virtual agents allow the learners recognise their emotions and understand how these affect the virtual agents and, subsequently, allow them to feel more empathy towards them.

## 2 Related work underlying the educational activity

Although it has been increasingly accepted that emotions are an important factor in improving human-machine interaction, many times digital teaching systems do not consider the emotional dimension that human users expect to encounter in an interaction, and this can lead to frustration (11). To be able to provide affective interaction between intelligent agents and human users, the computer system needs to provide the virtual agents with capabilities for emotional and socially intelligent behaviour which should, in real time, have a measurable effect on the user (15).

Emotion recognition has been widely explored in human-robot interaction (HRI) and affective computing (16). Recent works aim to design algorithms for classifying emotional states from different input modalities, such as facial expression, body language, voice, and physiological signals (17, 18). Ability to recognise human emotional states can encourage natural bonding between humans and robotic artifacts (19).

Andotra (20) found that by integrating emotional intelligence with technological capabilities, chatbots or virtual agents could enhance user engagement and well-being through personalised and empathetic interactions. To enhance conversational skills integrating emotional capabilities in chatbots is essential. AI-driven chatbots can detect user sentiments in a conversation, thus triggering the chatbot to comprehend the user's emotional state and generate an appropriate response (21).

There are several models for agent decision-making in interactive virtual environments, such as interactive healthcare teaching applications, for example:

• Belief, Desire, and Intention (BDI) (22), explained in more detail below
• Psi-theory (23), OCC (24) which models the human mind as an information processing agent, controlled by a set of basic physiological, social and cognitive drives
• and the Five factor Model (FFM), a grouping of five unique characteristics used to study personality: conscientiousness, agreeableness, neuroticism, openness to experience, extraversion.

Models are needed because agents are part of an environment that has limited resources, such as memory and computational power. This means that agents cannot take an infinite amount of time to process their next move(s). One of the most prominent models is the BDI model that uses a set of rules designed to reduce the number of possible actions the agent can perform, thus reducing the time needed for the agent to decide its next action. The environment within which the agents act changes continuously so the agent must decide upon its next move and act upon it quickly enough so that it ensures that the environment does not change in such a way that it may invalidate the selected action. BDI constrains the reasoning needed by the agent and, in consequence, the time needed for making a decision. Within BDI beliefs represent beliefs about oneself, other agents, and the world. Desires represent what the agent would like to achieve, such as situations it would like to accomplish. For example, an agent could desire a patient in a simulation to be helped with their meal. Intentions represent actions/plans the agent has committed to do.

Research on agent design architectures and emotions for virtual teaching of personnel for stressful situations focuses on emotion integration, the design of affective intelligent agents that interact with other agents (virtual or real) based on social norms and on how to improve agent realism and believability. Towards this goal some of the research combines and/or extends other theories and models.

For example, the extended BDI (EBDI) model (25), adds the influence of primary (first, more instinctive, emotional response to an event e.g., fear, sadness, anger) and secondary emotions (arising from higher cognitive processes and based on what we perceive that the result of a certain situation will be e.g., joy and relief). This makes agents more engaging and believable so that they can better play a human-like role in affective intelligent agent scenarios.

In addition to the user's emotions, virtual agents in our model (described in the next section—Architecture Overview) also adapt their actions and emotions based on the user's personality traits. The cognitive-behavioural architecture (26) incorporates the notion of personality as a determining factor for the agent's future emotions and coping preferences. This approach enables improved character diversity and personality coherence across different situations.

## 3 Design of the online learning environment

We present an architecture for creating a virtual tutor with the ability to alter both the tutor's and the virtual patient's behaviour and mood based on the responses, emotions, and personality of human participants. Responses to human participant's actions are selected from a database of possible rules that are based on previous research and input from psychologists and experts in the area of health training. The testing scenario used in this research focuses on teaching nurses caring for dementia patients.

The proposed architecture makes use of the BDI model and extends it by incorporating the emotions and personality of the trainees in the decision-making process. BDI is one of the most popular models for developing rational agents based on how humans act based on the information derived from an environment (27). BDI was chosen because it is one of the most prominent approaches to building agent and multiagent systems, it is based on a sound psychological theory, Michael Bratman's theory of human practical reasoning (28) and it has been successfully used for the design of realistic affective intelligent agents for many years (29–31).

The architecture proposed in this work extends BDI by incorporating two new elements. Firstly, it incorporates the current emotional state of the participant into its decision-making process. Systems that have explored emotion integration, such as the BDI tutor model (29) and Model Social Game Agent (MSGA) (30) have focused on just incorporating the emotions of trainees and do not take other factors into account, such as a human participant's personality traits. Our proposed model focuses on individual users and on the emotions of both the human participants and of the virtual agents. The emotional state of the human participant is not approximated by the model but is the dependant variable and reported by the participants in real time in order to convey the emotional state to the simulation for it to adapt. This introduces realism and personalises the process for the human participant as it focuses on the individual's emotional state and how this changes throughout the teaching session. With advances in artificial intelligence, virtual agents are starting to play an active role in various fields such as information presentation, sales, training, education, and healthcare (32, 33). To help induce a positive emotion and thereby enhance social bonding in human–virtual agent interaction, affective communication between people and virtual agents has become important (34).

Secondly, our architecture incorporates the personality of the participant. For the purposes of this research the Five Factor Model (also known as the “Big 5”) personality test (35) is used for measuring the participant's level of neuroticism. This has been suggested to be related to the level that negative emotions affect an individual during training (36–38). The feedback provided to the participant as well as the virtual patient's emotional state is affected by both these elements.

Regarding the reasoning rule-based model, the Jboss DROOLS Expert rules engine is used for providing feedback to the user and uses the adapted BDI model for providing emotional intelligence feedback. The front end of the learning environment has been designed using the UNITY3D game development system and programmed using C# to communicate with the server back end. The back end was programmed using JAVA and runs under Tomcat so that several front-end sessions can communicate concurrently with and between modules on the server.

### 3.1 Architecture overview

**Figure 1:**
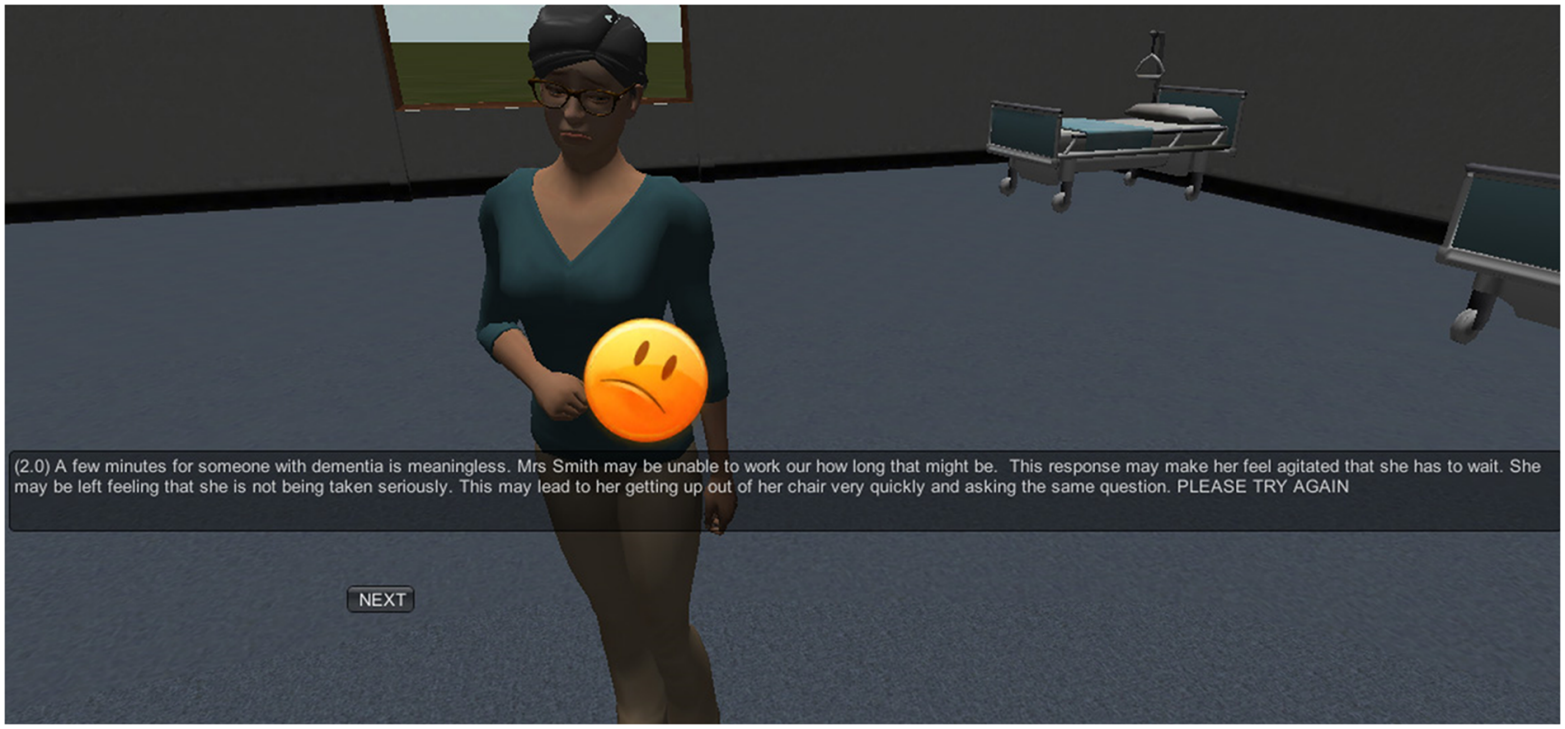
High-level overview of the proposed emotional virtual agent's architecture.

A high-level overview of the emotional virtual agent's architecture can be seen in Figure 1. The architecture comprises 4 sections. The “User” section illustrates the real world and the participant taking part in the virtual teaching simulation. The “Agent Interface” section illustrates the communication between the participant and the virtual agent. The “Cloud Communication” section illustrates the data communication between the virtual teaching environment and the emotional agent architecture on the server. Finally, the “Evaluation” section illustrates the decision-making process based on the BDI system, available plans, human participant emotions and personality. Once inputted, the events, emotions and personality characteristics of the participant are sent from the virtual learning environment to the server, as shown in the “Cloud Communication” section.

The interaction between the participant and the agent interface, shown in the “User” and “Agent Interface” sections of Figure 1, is performed using traditional input devices, such as the keyboard and mouse. The participants take part in an interactive 3D virtual teaching scenario. The communication between the participant and the virtual tutor/patient is achieved using dialog boxes. The virtual tutor has no embodiment within the virtual environment. It presents messages and feedback to the participant via text boxes. The virtual patient is represented within the virtual environment by a 3D human model. They are controlled using scripted behaviours; however, the virtual tutor can alter the selection of behaviour and the mood based of the virtual patient based on the responses, emotions, and personality of human participant.

**Figure 2:**
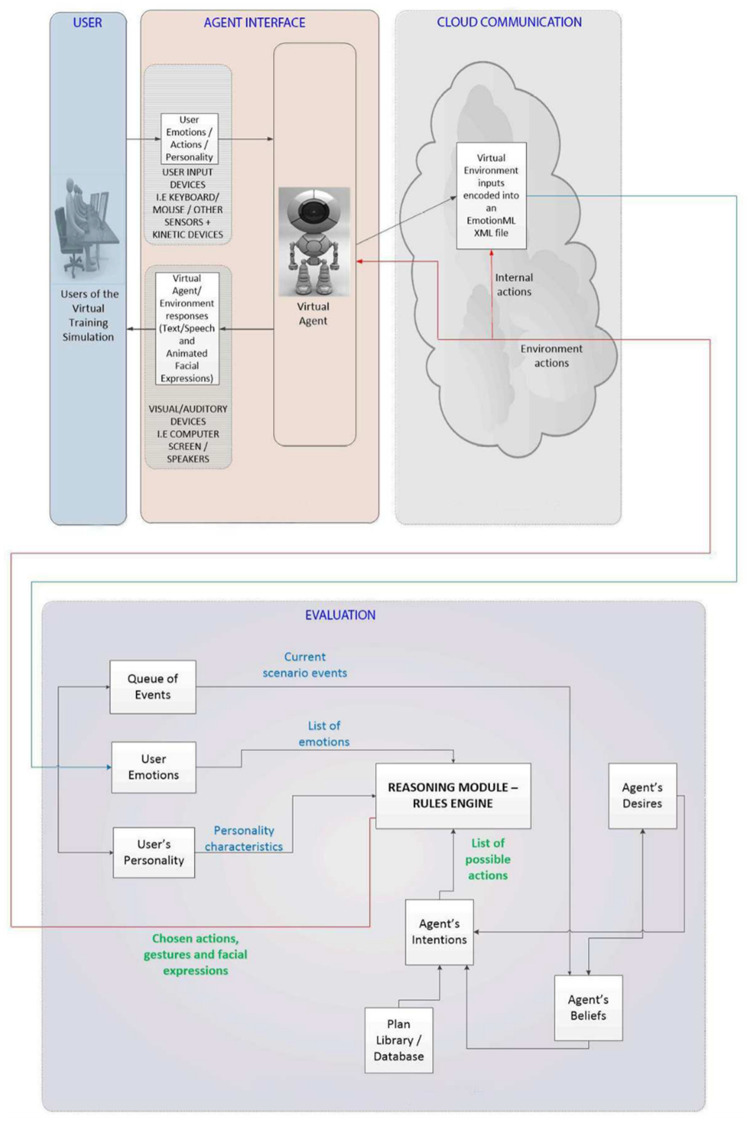
A screenshot of the virtual teaching environment and an interaction with the virtual agent. Please enter a scale for the emotion Stress and then reply to the question below—Current Value 0; Please enter a scale for the emotion Agitation and then reply to the question below—Current Value 0; Please select Emotions; (Q1 of 5) Should I be here in this place?:- I know you are worried about being here in hospital. Did you need to use the toilet?; You are in hospital and you have to stay in for quite some time; Please stop talking to me and interrupting me while I am trying to do the medicine round.

The participants have a first-person view of the virtual teaching environment and select their choice of action using selection boxes, as shown in Figure 2. Participants input their personality type and their current emotional status by selecting the type and intensity of their emotions, using selection grids. This method of emotional input is used as it is a quick and reliable way for collecting information on the emotional status of a trainee in a real time environment.

### 3.2 Adapting the BDI model

The BDI model is used as the basis for interactive virtual environment decision-making in the emotional virtual agent's architecture. The virtual tutor's beliefs are created and updated based on its desires and current scenario events. The tutor uses dialogue boxes for teaching the participants and prompting them to perform different actions. The participant responds by performing the required actions and replies to the tutor using multiple choice and true/false responses.

As illustrated in Figure 1, the decision-making process consists of the following modules:

• The agent's Beliefs module accepts as input events from the current scenario and the agent's desires. Based on these inputs, the module outputs a list of the agent's beliefs to the agent's desires and intentions modules. For example, a scenario input could be a virtual patient that needs help finishing his meal and the tutor's desire to provide the needed help to the patient. The outputs of the module would include the intention to ask a participant to help the patient and the desire to follow through by confirming at a later time that the participant fulfilled her task.
• The agent's Desires module accepts as input a list of the agent's beliefs and outputs a list of the agent's desires to both the agent's beliefs and agent's intentions modules. Based on the example illustrated above, the module could receive the desire of the agent to confirm that the participant helped the virtual patient finish his meal. The module would then output the intention of asking the participant at a later time if she fulfilled the task of helping the virtual patient and if the patient did, indeed, manage to finish his meal.
• The agent's Intentions module accepts as input a list of possible plans, the agent's desires and the agent's beliefs. It outputs a list of possible actions to the reasoning module. For example, inputs could include the plan of the tutor to ask the participant to help the virtual patient finish his meal by helping him sit at the table and cut the meat in manageable pieces, the desire of the tutor to help the patient and the belief that the patient needs the participant's help for finishing his meal.
• The agents Reasoning Module accepts as inputs a list of possible participant responses to the tutor's question and the participant's emotions and personality traits. It outputs the chosen actions to be communicated to the participant and a set of gestures and facial expressions to be used by the virtual patient's 3D avatar. For example, if a participant's inputs indicate that they are tired and not very empathetic then the tutor would ask her in a firmer way to help the virtual patient. The tutor would be less firm when communicating with a rested participant that is more likely to feel empathy for the patient that has difficulty finishing his meal.

### 3.3 Incorporating emotions and personality into belief–desire–intention model

Participant emotions and personality in the proposed architecture are inputted by the participant using the keyboard/mouse within the virtual learning environment. These are then transferred over a network to the server/back-end to be interpreted by the inference engine. The participant's input their emotions and personality by replying to multiple choice questions, selecting one or more emotions and their intensity (using a Likert scale). As discussed above, for this research the Five Factor Model (also known as the “Big 5”) personality test (35) is used for measuring the participant's level of neuroticism.

The participant's personality and emotions and the virtual agent's desires, intentions and beliefs are the inputs to the emotion creation and revision module. Emotions affect the best possible way an agent can respond to his requests. Personality affects the intensity and duration of these emotions. For example, using the Myers Briggs Personality Types, a Facilitator Caretaker incorporating the following Myers-Briggs Personality Preferences: Extraversion Sensing Feeling Judging, is more likely to feel compassion and sadness for a patient than a Conceptualizer Director that incorporates the following preferences: Introversion, iNtuition, Thinking, Judging.

The virtual agents in our model predict how to respond to the indicators of a participant's emotions depending on an inference of the participant's personality. The participant's neuroticism level is used then to adjust the scale grade of the reported emotion. As people with higher levels of neuroticism tend to over-report their negative emotions, the rule is to decrease the level of emotion reported on an 1–10 scale from participants with higher than average levels of neuroticism and lower the reported level for participants with lower than average levels of neuroticism.

### 3.4 The reasoning module

A set of rules adapts the responses of the virtual tutor based on the trainee's emotions, emotion strength, and personality traits.

**Figure 3:**
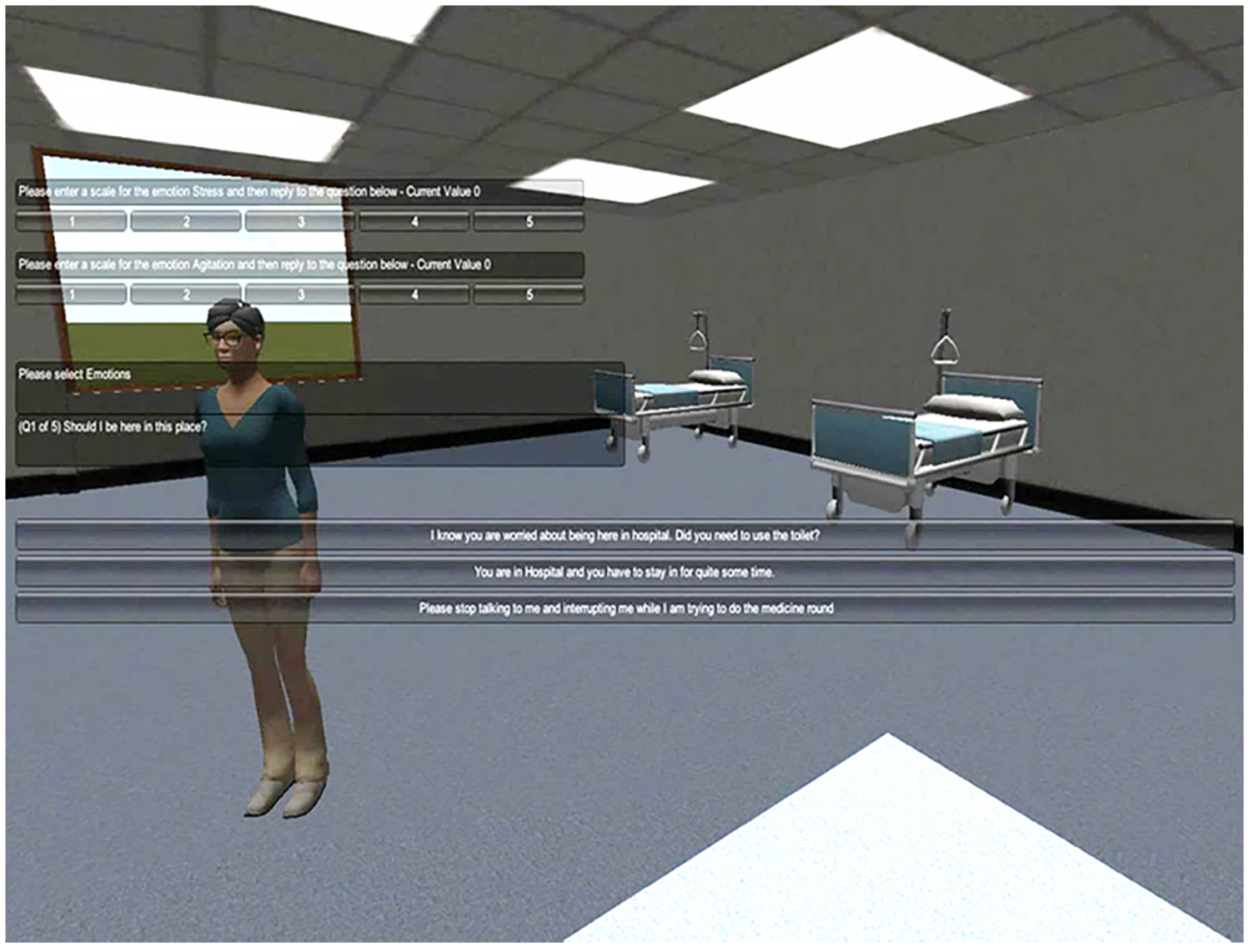
User of animations, sounds and emoticons for portraying the emotion of sadness for the virtual patient. “A few minutes for someone with dementia is meaningless. Mrs Smith may be unable to work out how long that might be. The response may make her feel agitated that she has to wait. She may be left feeling that she is not being taken seriously. This may lead to her getting up out of her chair very quickly and asking the same question. PLEASE TRY AGAIN.”

Our system outputs the feedback to be given to the participant, and information that identifies how the participant's response affected the emotional state of the virtual patient. This information can then be used to adjust the virtual patient's behaviour and animations. For example, an animation can be played, and an emoticon can be displayed that show the patient's resulting emotional state. This approach allows the system to decide which actions will be performed by the virtual agent and what types of gestures, facial expressions and emoticons will be used for interacting with the participant within the virtual teaching environment. In a similar manner, depending on the virtual patient feedback, which is directly related to the emotion felt by the patient, an emoticon is displayed on the screen that shows clearly how the patient feels. The possible emotions portrayed are happiness, sadness, disgust, fear and anger. Figure 3 illustrates an example of sadness for the virtual patient.

## 4 Results

The chosen scenario for the data collection was designed by a team of nursing tutors that specialise in mental health and, specifically, in treating patients with dementia. The participants for the case studies consisted of nine nursing students with an interest in mental health. The data collection consisted of:

1. A pre-study interview that gathered background information about the participant;
2. An online personality test (Five Factor Model). This was used to determine the level of neuroticism of the participants;
3. Two teaching sessions. During the sessions, the participants interacted with a virtual patient with dementia and responded to common questions asked by such patients. During the interaction, interruptions occurred that had to be dealt with (these were random in occurrence and duration, in the same way these would occur in the real world), such as another patient asking for water or the fire alarm going off. These interruptions were chosen based on input from experienced nurses. The training comprised two different scenarios:
  a. Scenario 1—A teaching scenario where the feedback given to the participants did not depend on their emotions and personality and the patient did not demonstrate any visual or auditory change in their mood. This session was delivered as a point of reference to the participant on how systems that do not cater for different personalities and emotions work;
  b. Scenario 2—an updated system based on the model designed for this research; the second scenario provided visual (emoticons and animations) and auditory feedback on the patient's emotional state. These were based on the trainee's emotions, personality, and responses to the patient's questions.
4. An interview was conducted for gathering data on how the teaching session affected the learning process, emotional states and general satisfaction of the participants. Interview questions (Appendix A) were semi-structured and the participants were prompted to discuss and expand their replies.

A set of qualitative data was collected using observation and semi-structured interviews. The data was used to draw out patterns of how visual and auditory representations of a virtual patient's mood may affect the learning process, emotional states, and general satisfaction of the trainees.

• **Data analysis**

This section presents and interprets the qualitative results from the testing of the developed emotional virtual agent's architecture.

The participants were selected using convenience sampling from a 3-year nursing course at a university. Students were in their early 20s, 7 were female and 2 male, a representative ratio of nursing students according to statistics (39). Inclusion criteria included that the participants were taking part in their hospital placements in the mental health ward and were working with patients with dementia. The study was approved by the university's ethical committee and the participants signed a consent statement before participating. The sample size was deemed to be large enough for a small-scale qualitative test of the technology to evaluate feasibility and acceptability. A future quantitative study with a larger number of participants will be used to evaluate statistical significance.

A summative content analysis was first used to identify themes related to the research objectives. In the table (Appendix B) the number of occurrences that support each research theme for each of the nine participants are listed. The research objectives (RO) are as follows:

• RO1—To what extent does a virtual learning scenario incorporating emotional virtual agents provide a realistic experience.
• RO2—To what extent does the incorporation of emotions into virtual agents stimulate a better set of responses from the human user.
• RO3—To what extent do emotional virtual agents improve the learning experience.
• RO4—To what extent do emotional virtual agents allow the learners recognise their emotions and understand how these affect the virtual agents and, subsequently, allow them to feel more empathy towards them.

Below is a summary of the occurrences for each RO from interviewing the participants

Table 1 illustrates how the scenario that provided text responses that were affected by the participant's personality traits and emotions, in addition to using visual and auditory cues, was more successful in providing a more realistic experience, better responses, improved learning and increased empathy.

**Table 1:** Summary of the occurrences for each RO from interviewing the participants.

Theme Description	Realistic experience	Better responses	Improved Learning Experience	Increased Empathy
Interview occurrences Participant 1	4	4	4	6
Interview occurrences Participant 2	4	3	3	5
Interview occurrences Participant 3	2	3	3	3
Interview occurrences Participant 4	2	2	2	2
Interview occurrences Participant 5	1	1	2	2
Interview occurrences Participant 6	2	3	1	1
Interview occurrences Participant 7	7	2	1	2
Interview occurrences Participant 8	3	2	3	4
Interview occurrences Participant 9	1	1	4	3

In more detail, Increased Empathy was the research objective that had the highest number of occurrences. This is a very important part of what we tried to achieve with our VR scenario educational design as we aimed to have a patient-centered educational design, something extremely important in general, but even more in the mental health sector, The next RO with 26 occurrences was Realistic Experience, something that a VR experience can certainly provide if designed correctly. Improved learning experience and Better Responses to patient questions followed closely with a still high 23 and 21 occurrences respectively, showing that all of the ROs were sufficiently achieved.

In the remainder of this section, we will provide a detailed analysis of the qualitative data collected for this study. The analysis is organised according to the research objectives outlined at the start of this section.

• **To what extent does a virtual learning scenario, incorporating emotional virtual agents provide a realistic experience**

All participants (*n* = 9) reported that the scenario questions were realistic, based on their experience of roleplaying teaching and actual first-hand experience of working with people with dementia during hospital placement, and made them feel that they were interacting with a real patient, especially in the case that the patients reacted in a way that made their emotions clear. They also felt that the realism of the scenario could help them improve their nursing skills, especially with the integrated distractions which corresponded to what they would expect to have to deal with in a real-world scenario. They found that the realism of the system could provide a good alternative to training with real people either at university or during their placements. The realistic sounds from the patients (laughter, screams etc.) were also found to be very useful and increased the realism of the experience.

One participant stated in the initial interview that took place before the teaching session that he believed that online teaching would never replace practicing with real people.

“I’m a firm believer that simulated and online experiences will never replace the total quality of experiencing that in practice.” (Participant 9).

After the session the same participant reported that he found the online teaching to be much more realistic than expected:

“I think that's exactly the kind of challenges that a healthcare worker will have. They will go into a bay, they’ll be approached by one patient but there will be others also demanding on their time, there’ll be other important tasks and it's important to make sure that the patient is cared for and you also meet the needs of others. So, the scenarios felt very real to me.” (Participant 9).

• **To what extent does the incorporation of emotions into virtual agents stimulate a better set of responses from the human user**

All the participants reported that the emotionally enhanced teaching scenario made them realise how their actions can affect the patient's mood and subsequent reactions and how by being calm and not letting their emotions guide their actions they can help the patients much more. They also agreed that seeing how happy the patient was because of their care also improved their mood. The affective virtual agents also made the participants reflect on their work, when the results were positive but, mostly, when they did not succeed in keeping the patient happy. For example, one participant stated:

“… in one of the questions the patient was shouting, he wanted to get out of there. If I was shouting back it would have escalated, it would have made the patient more angry, whereas if I just calmly say we can help then at least the patient's hear that there is some help for them.” (Participant 1).

They continued to say that reporting these emotions helped him try to control them:

 “… so I’ve got to keep it in and just find an easier, calmer way to say, ‘Let's sit down and we’ll sort it out.’” (Participant 1).

The participant also said that asking him to report these emotions helped him think about how he felt and prompted him to try and choose the answer that would make it less likely to transfer his negative emotions onto the patient.

“Yeah, because there were some answers that I was reading, and I thought if I answer with that it will make the scenario escalate and make the patient more agitated and angry if I tell them, “Oh just sit down, I’ll deal with you later.” They don’t feel like they’re being cared for, so they’ll just want to leave, they won’t want to sit down and talk to you but if you say, ’Sit down, I’ll get you a drink and we’ll talk about how worried you are.’” (Participant 1).

The online teaching environment also helped them respond better as they felt more secure not having to deal with real patients while still at the initial stages of learning as the embarrassment of getting one question wrong in the real-world would sometimes make them get more wrong answers.

• **To what extent do emotional virtual agents improve the learning experience**

Again, all the participants stated that the feedback based on their responses made them understand clearly how their answers affected the patient and it allowed them to better understand how important it is to know how the patient feels at every stage of their interaction. They reported that the experience seemed realistic and close to what they would expect to do in real life, and this helped them learn better. It was also something they found more enjoyable and less stressful as real-life teaching scenarios sometimes made them feel uncomfortable and as they were judged by their tutor. They felt that these teaching sessions within a “safe” environment would allow them to practice and improve their skills before attempting to work with a real patient. For example:

“So, like when we did it face to face with a member of staff in a way you could feel that you were being judged by that member of staff. I suppose to test out your skills first online then you’re sort of in a safer environment than when you’re face to face with somebody. Learning this online is more of a safe environment to get it wrong and where you can get your skills up to scratch really to be able to do it face to face with somebody.” (Participant 3).

The teaching sessions also made them realise that this kind of teaching can also be applied to other patients with different conditions where it would also help improve the learning experience. Responses included:

 “I think that's the thing that's missing in practice is that nobody tells you, you are so driven within the moment of stress that you don’t realise you are and you do need to take a step back. I think that's perhaps the difference here between the virtual environment and the real environment, there is no button in the real environment to press, to gauge my anxiety or my stress, to help me realise it, to help me control it.” (Participant 9).

• **To what extent do emotional virtual agents allow the learners recognise their emotions and understand how these affect the virtual agents and, subsequently, allow them to feel more empathy towards them**

The extent to which affective virtual agents allow carers to increase their empathy was investigated. Both the replies to the interview questions and the observation from the researcher provided data that supported the research objective. More specifically the participants reported that they realised how patients can feel threatened when carers let their negative emotions show and, on the other hand, how by seeing a patient being calm and happy they know that they succeeded in giving them the right care and they did not let their stress, anger or other emotions affect their work. For example, one participant commented on how having to report how stressed and agitated he was before every reply to a question helped him understand better how those emotions could affect the patient.

“Well, it made me really think of the patient. That it could escalate the patient's condition and make him feel threatened if the trainee let those emotions show.” (Participant 1).

In the same way he noted that reporting those emotions help him think about them more, and subsequently made him try to control them.

“Yeah, if I’m shouting, even though I might feel angry and just want to finish, if I show that it might make the patient feel threatened.” (Participant 1).

He also stated that asking him to report those emotions helped think about how he felt and try to choose the right answer that wouldn't affect the patient negatively.

“Yes, you may not have the time to and you may need to do other stuff but that way at least you’ve calmed the patient down a little.” (Participant 1).

The reactions of the patient in the second scenario made them realise clearly how the patient felt and made them try even more to be compassionate and calm and not allow their actions to have a negative impact on the patient. All the participants agreed that a happy patient made them feel happy and contented too whereas a distressed patient affected them negatively. Responses included:

“Yes, because if you feel you’re affected emotionally by the situation you do think about what it is you’ve got to say next and what you’re going to do next because you know that you don’t want your emotions to impact on the patient because it's not about you, it's about the patient and that side of care.” (Participant 4).

The results of the comparison between the two different systems are outlined below. The system that provided the visual and auditory representations of the virtual patient's emotions based on the participant's emotions, personality and responses was reported by the nice participants to have the following advantages:

1. Provided a more realistic representation of the carer/patient interaction;
2. Performed better in helping the carers:
  a. recognise how the patients feel;
  b. recognise and evaluate their own emotions;
  c. realise how their actions can affect the patient's emotional state;
  d. realise how their emotions can affect the patient's emotional state;
  e. empathise with the patients.

To summarise, all (100%) of the participants reported, when using the enhanced scenario, a more realistic representation of carer/patient interaction; better recognition of the patients' feelings; recognition and assessment of emotions; a better realisation of how feelings can affect patients' emotional state and how they could better empathise with the patients.

## 5 Discussion

Our aim was to explore how intelligent virtual agents in healthcare provision teaching simulations can improve the human participant's learning experience by incorporating visual and auditory representations of their emotional states. These emotional states and the virtual agent's responses were adapted based on an indication of the participant's emotions and personality.

We found that visual and auditory representations of the patient's emotional state based on our adapted BDI architecture positively affected the learning process, emotional states, and general satisfaction of trainee nurses. Two scenarios were used; one that did not include a virtual patient with different mood states and an updated system that provided visual and auditory feedback based on the patient's emotional state.

There was no negative feedback regarding the second, emotionally enhanced, scenario. When completing the emotionally enhanced scenario some of the participants even had a complete change in heart regarding the realism and usefulness of online learning environments. All the nurses reported that the second scenario using the enhanced learning system was more realistic, helped them better realise how their actions and emotions can affect a patient and, thus, made them more empathetic and improved their learning experience.

Rich data was collected from the nine participants that support that the adapted scenario with the emotionally enhanced patients allowed them to:

• Experience a more realistic representation of carer/patient interaction
• Better recognise the patients' feelings;
• Better realise how feelings can affect patients' emotional state
• Empathise with the patients.

These results will be useful for researchers in designing and conducting future studies relevant to a broader range of healthcare providers. By creating more varied scenarios focusing on different target groups, e.g., people with learning disabilities, people that suffered from stroke etc., the architecture can be tested further and modified, as/if necessary, to better cater for different health conditions.

Previous research in this area has focused on:

1. Non interactive videos where the participants did not have a hands-on experience, as we mentioned in the introduction of this paper regarding the systematic review of nursing education teaching technologies (6).
2. scenarios focusing on only the emotions of the participants and not on both their emotions and their personality (40, 41).

Our work can be further adapted to be used in different and more varied fields, including crisis management and with both formal (soldiers, firefighters, law enforcement officers) and informal personnel. This can be done by using a different set of emotions for the participants; these can be negative, positive or include some of each type of emotion depending on the field and scenarios used. In future work, we plan to incorporate online assessments instruments into the virtual scenario for collecting quantitative data on how emotionally enhanced virtual agents could improve the learning experience.

## Data Availability

The raw data supporting the conclusions of this article will be made available by the authors, without undue reservation.

## Appendix A: Questions from the semi-structured interviews

**Table:**

**PART 1: Before completing the scenarios in the online environment**
1. Please tell me a bit about yourself, what year you are in your training, and your main areas of interest?
2. What particular challenges do you face in your training when working with patients?
3. Do you feel that online training is something that could help you improve working with patients?
4. Have you done any online nursing before and, if yes, how did you find it?
5. Have you done any training with real actors before and, if yes, do you prefer training with real actors or virtual patients?
**PART 2: After completing the scenarios in the online learning environment**
1. How did you find them easy or difficult to go through? Please explain.
i. Did these feel realistic:
a. 3D environment. Please explain. b. Scenario/Questions. Please explain
2. Did reporting your emotions during the scenario help you understand better how these emotions can affect the patient? Please explain.
3. Did reporting your emotions during the scenario help you in thinking about and, subsequently, trying to control your emotions? Please explain.
1. Did the changes in the patient's mood in scenario 2 affect your feelings during the scenario? Please explain.
2. Did the changes in the patient's mood in scenario 2 make it clearer how your replies affected her? Please explain.
3. Did you find any of the two scenarios to:
a. Be more realistic. Please explain. b. Improve your learning more. Please explain. c. Make you more empathetic towards the patient. Please explain. d. Work better for you in any other way. Please explain.
4. What changes would you suggest we make to future online training scenarios?
5. Do you feel that more similar training could help you improve your nursing skills further? Would it help you become even more compassionate with patients? Please explain.
6. Would you be interested in taking part in more online training scenarios in the future?

## Appendix B

Themes table

Themes related to the research objectives

**Table:**

Theme Number	Code	Theme
01	REAL-EXP	Realistic experience (RO1)
02	BET-RESP	Better responses (RO2)
03	IMP-LEXP	Improved Learning Experience (RO3)
04	INC-EMP	Increased Empathy (RO4)

Themes related to the Participant 1 (P1)

**Table:**

# of citations	Code	Theme
4	REAL-EXP	Realistic experience (RO1)
4	BET-RESP	Better responses (RO2)
4	IMP-LEXP	Improved Learning Experience (RO3)
6	INC-EMP	Increased Empathy (RO4)

Themes related to the Participant 2 (P2)

**Table:**

# of citations	Code	Theme
4	REAL-EXP	Realistic experience (RO1)
3	BET-RESP	Better responses (RO2)
3	IMP-LEXP	Improved Learning Experience (RO3)
5	INC-EMP	Increased Empathy (RO4)

Themes related to the Participant 3 (P3)

**Table:**

# of citations	Code	Theme
2	REAL-EXP	Realistic experience (RO1)
3	BET-RESP	Better responses (RO2)
3	IMP-LEXP	Improved Learning Experience (RO3)
4	INC-EMP	Increased Empathy (RO4)

Themes related to the Participant 4 (P4)

**Table:**

# of citations	Code	Theme
2	REAL-EXP	Realistic experience (RO1)
2	BET-RESP	Better responses (RO2)
2	IMP-LEXP	Improved Learning Experience (RO3)
2	INC-EMP	Increased Empathy (RO4)

Themes related to the Participant 5 (P5)

**Table:**

# of citations	Code	Theme
1	REAL-EXP	Realistic experience (RO1)
1	BET-RESP	Better responses (RO2)
2	IMP-LEXP	Improved Learning Experience (RO3)
1	INC-EMP	Increased Empathy (RO4)

Themes related to the Participant 6 (P6)

**Table:**

# of citations	Code	Theme
2	REAL-EXP	Realistic experience (RO1)
3	BET-RESP	Better responses (RO2)
1	IMP-LEXP	Improved Learning Experience (RO3)
1	INC-EMP	Increased Empathy (RO4)

Themes related to the Participant 7 (P7)

**Table:**

# of citations	Code	Theme
7	REAL-EXP	Realistic experience (RO1)
2	BET-RESP	Better responses (RO2)
1	IMP-LEXP	Improved Learning Experience (RO3)
2	INC-EMP	Increased Empathy (RO4)

Themes related to the Participant 8 (P8)

**Table:**

# of citations	Code	Theme
3	REAL-EXP	Realistic experience (RO1)
2	BET-RESP	Better responses (RO2)
3	IMP-LEXP	Improved Learning Experience (RO3)
4	INC-EMP	Increased Empathy (RO4)

Themes related to the Participant 9 (P9)

**Table:**

# of citations	Code	Theme
1	REAL-EXP	Realistic experience (RO1)
1	BET-RESP	Better responses (RO2)
4	IMP-LEXP	Improved Learning Experience (RO3)
3	INC-EMP	Increased Empathy (RO4)
